# Special feature on evolution and genetics in medicine

**DOI:** 10.1098/rspb.2015.2811

**Published:** 2015-12-22

**Authors:** Brian G. Spratt, Roy M. Anderson

**Affiliations:** Department of Infectious Disease Epidemiology, Imperial College London, London, UK

The origins of genetics and evolution are relatively recent in the context of the 350 years of scientific publication that are celebrated this year by the Royal Society, emerging in the 1850s and 1860s with the work of Gregor Mendel and Charles Darwin. The rediscovery of Mendel's laws of inheritance in 1900, and the demonstration by Garrod [1] in 1902 that they could explain the familial distribution of alkaptonuria, was perhaps the first contribution of genetics to medicine.

Important work in the 1920s and 1930s by Haldane, Fisher and Wright on the development of population genetics and, subsequently, the formulation of the modern evolutionary synthesis provided the basis of our current understanding of population and evolutionary biology. Haldane was also probably the first to introduce an evolutionary perspective into medicine, suggesting in 1949 that selection for resistance to malaria might explain the high levels of thalassaemia in malarious regions [2].

However, the discoveries that led most directly to the remarkable recent progress in genetic and evolutionary approaches in medicine go back to the 1940s and 1950s with the establishment of DNA as the genetic material and the elucidation of its structure, with the subsequent emergence of molecular biology in the following decade.

An important recent stimulus to research on genetics and evolution in medicine was the sequencing of the human genome. On 26 June 2000, the International Human Genome Sequencing Consortium announced the production of a rough draft of the human genome sequence. In April 2003, the Consortium announced an essentially finished version of the human genome sequence. This version, which is available to the public, provided nearly all the information needed to research almost all aspects of the whole genome. The initial promise of this achievement, for rapid progress in understanding the genetic basis of many common human diseases, was slow to be realized given the polygenic nature of the susceptibility of individuals to most of these conditions.

However, in recent years, progress has been steady, due to new methods for rapid and low-cost sequencing of multiple genomes, combined with advances in bioinformatics and new statistical tools that permit the identification of complex multi-genic associations between host genetic background and the likelihood of a particular disease occurring. Concomitant with advances in the study of the human genome has been the complete sequencing of all the major pathogens of humans, and the use of these sequences to address a wide range of problems, including defining ‘who infects whom’, and what genetic factors control pathogenicity and transmissibility. Complete sequences for the human host and a particular infectious agent have revealed the complex interplay between the two genetic templates in all host–parasite associations in determining the course of infection and associated morbidity and mortality.

In this issue of the *Proceedings of the Royal Society B: Biological Sciences*, six contributions are presented that chart past and present progress in a series of important fields where genetic and evolutionary research have made important contributions to a variety of areas in medicine. The areas covered are not exhaustive of the fields where such research has made important contributions to current medical practice and the treatment of disease. For example, one very important area not covered in this issue is cancer research, where the ability to routinely sequence the genomes of tumour cells is already having both a major clinical impact and an impact on the design and testing of new small molecule or immunotherapeutic treatments. However, the contributions in this issue provide an exciting reflection of current genetic and evolutionary research, which is advancing our understanding of the causes and treatment of disease and infection.

Current progress in genome-wide association studies between genetic polymorphisms and common genetic disorders is reviewed by Donnelly and co-workers [3]. Technological advances have allowed the detection of genome-wide genetic variation in large population cohorts, which, combined with sophisticated statistical analyses, are allowing the identification of variants associated with susceptibility to common genetic diseases. These studies provide the identification of genes and pathways that may lead to a better understanding of these diseases as well as new approaches to treatment.

Collins & Thrasher [4] review progress in gene therapy, another area that has been a focus of much recent research, although progress has been relatively slow. However, new approaches, including the ability to precisely correct genetic defects—gene editing—promise major advances in gene therapy, although ethical concerns still have to be addressed.

The origins of immunology can be traced back to at least the early eighteenth century, but studies during the last 60 years have provided a detailed picture of the complex systems that have evolved to protect against disease. The article by McMichael and co-workers [5] focuses on what is known about the development and decline of the human immune system from birth to old age, and the consequences for protection against infection and for autoimmunity and malignancy.

Cardiovascular diseases remain a major cause of morbidity and mortality throughout the world, and Kennedy-Lydon & Rosenthal [6] discuss the prospects for cardiac regeneration to repair the damage resulting from mycocardial infarction.

A very different area where large-scale genome sequencing is having an impact is in the study of pathogens, where sophisticated analytical tools that combine the genome sequences of large samples of pathogen isolates with geospatial and temporal data are leading to an understanding of the origins, evolution, pathogenicity and epidemiology of viral and bacterial diseases, with the prospect of greatly improved real-time surveillance of disease outbreaks. Pybus *et al*. [7] and Bentley & Parkhill [8], respectively, describe recent progress in this area for viral and bacterial pathogens.

We hope this selection of contributions will serve as a stimulus for expanded research in this fascinating field that combines old (but fundamental) concepts such as the theory of evolution by natural selection, with new technologies in whole-genome sequencing and molecular biology that are beginning to unravel the detailed mechanisms of disease causation and highlight possible new avenues for treatment or cure.

## References

[1] Garrod AE 1902 The incidence of alkaptonuria: a study in chemical individuality. Lancet 160, 1616–1620. (10.1016/S0140-6736(01)41972-6)

[2] Haldane JBS 1949 The rate of mutation of human genes. Hereditas 35, 267–273. (10.1111/j.1601-5223.1949.tb03339.x)

[3] Price AL, Spencer CCA, Donnelly P 2015 Progress and promise in understanding the genetic basis of common diseases. Proc. R. Soc. B 282, 20151684 (10.1098/rspb.2015.1684) PMC4707742 26702037

[4] Collins M, Thrasher A 2015 Gene therapy: progress and predictions. Proc. R. Soc. B 282, 20143003 (10.1098/rspb.2014.3003) PMC4707739 26702034

[5] Simon AK, Hollander GA, McMichael A 2015 Evolution of the immune system in humans from infancy to old age. Proc. R. Soc. B 282, 20143085 (10.1098/rspb.2014.3085) PMC4707740 26702035

[6] Kennedy-Lydon T, Rosenthal N 2015 Cardiac regeneration: epicardial mediated repair. Proc. R. Soc. B 282, 20152147 (10.1098/rspb.2015.2147) PMC4707759 26702046

[7] Pybus OG, Tatem AJ, Lemey P 2015 Virus evolution and transmission in an ever more connected world. Proc. R. Soc. B 282, 20142878 (10.1098/rspb.2014.2878) PMC4707738 26702033

[8] Bentley SD, Parkhill J 2015 Genomic perspectives on the evolution and spread of bacterial pathogens. Proc. R. Soc. B 282, 20150488 (10.1098/rspb.2015.0488) PMC4707741 26702036

